# Supplementary material for: Timely bespoke phage-antibiotic combination to treat refractory Pseudomonas aeruginosa mediastinitis and vascular graft infection
Source: Nat Commun. 2026 Jan 9;17:1385. doi: 10.1038/s41467-025-68136-y (PMC12877064; doi:10.1038/s41467-025-68136-y)
Supplement: Supplementary file 1 — Supplementary Information [file 41467_2025_68136_MOESM1_ESM.pdf]

Supplementary Information

Table of Contents

1. Therapeutic drug monitoring (TDM) 3

a. Methods for therapeutic drug monitoring 3

b. Results of therapeutic drug monitoring 3

2. Synergy E-test. 6

a. Methods of synergy E-test 6

b. Results of synergy E-test 6

3. Susceptibility profile of the patient's clinical P. aeruginosa isolates 6

a. Methods 6

b. Interpretative categories and disc diffusion zone diameters of four individual *P. aeruginosa* isolates based on Clinical Laboratory Standards Institute (CLIS) methods 6

c. Minimum Inhibitory Concentrations (MICs) of four individual *P. aeruginosa* isolates and a four-bacteria mixture using microtiter MIC methods 8

4. Methods and results of whole genome analysis of the P. aeruginosa isolates 10

a. Methods 10

b. Results 10

5. Phage genomic profiles 13

6. Transmission electron microscopy (TEM) images of 8 candidate therapeutic phages 18

7. Identification of suitable therapeutic phages 19

a. Phage susceptibilities via spot test 19

b. Antibiotics Micro-titre MICs assay when combined with phages 20

c. Kill kinetic studies of phages against patient clinical isolates 22

d. Antibiotics kill kinetics against 4 patient clinical isolates combined 27

e. 3-Phage cocktails + antibiotics kill-kinetic against 4 patient isolates combined 28

f. Single-phage and cocktail kill-kinetic at low MOI 1 against 4 patient clinical isolates combined. 30

8. Anti-Biofilm Activity 31

9. Role of the MexAB-OprM efflux pump in phage susceptibility 32

10. Single-phage escape bacteria mutant cross-resistance profiles 33

11. Detection of Therapeutic Phages in Patient Blood 35

Supplementary references 39

Inventory of Tables and Figures in Supplementary Information

SUPPLEMENTARY TABLES

Table S1. Results of therapeutic drug monitoring
Table S2. Interpretative categories and zone diameters of *P. aeruginosa* isolates based on CLSI methods
Table S3. Minimum inhibitory concentrations (MICs) of four *P. aeruginosa* isolates and a four-bacteria mixture using microtiter MIC methods
Table S4. Results of whole-genome analysis of the four *P. aeruginosa* isolates
Table S5. Summary of genomic information of the three therapeutic phages
Table S6. Phage genomic passport of PW21
Table S7. Phage genomic passport of KSY1a
Table S8. Phage genomic passport of P0413
Table S9. Bacteria phage–antibiotic susceptibility profiles
Table S10. Cross-resistance profile of single-phage escape mutants
Table S11. PCR target regions, expected amplicon sizes, and primer sequences for phage detection

SUPPLEMENTARY FIGURES

Figure S1. Defence system presence/absence matrix of clinical *P. aeruginosa* isolates
Figure S2. TEM images of candidate therapeutic bacteriophages
Figure S3. Spot-test susceptibilities of *P. aeruginosa* isolates to 8 phages
Figure S4. Time-kill kinetics of isolate PA2081 with single phages
Figure S5. Time-kill kinetics of isolate PA2091 with single phages
Figure S6. Time-kill kinetics of isolate PA2096 with single phages
Figure S7. Time-kill kinetics of isolate PA2111 with single phages
Figure S8. Time-kill kinetics using 2-phage cocktails
Figure S9. Time-kill kinetics using 3-phage cocktails
Figure S10. Antibiotic-only time-kill kinetics
Figure S11. Time-kill kinetics of cocktail 1 + antibiotics
Figure S12. Time-kill kinetics of cocktail 2 + antibiotics
Figure S13. Time-kill kinetics of cocktail 3 + antibiotics
Figure S14. Time-kill kinetics of cocktail 4 + antibiotics
Figure S15. Time-kill kinetics at low MOI (MOI = 1)
Figure S16. Biofilm dispersion assays with phage cocktails
Figure S17. Role of MexAB-OprM efflux pump in phage susceptibility
Figure S18. PCR detection of therapeutic phages in patient blood
Figure S19. PCR optimisation results for phage detection

1. Therapeutic drug monitoring (TDM)

a. Methods for therapeutic drug monitoring

Blood samples were collected to capture peak and trough levels, whenever feasible for antibiotics on intermittent dosing. For continuous antibiotic infusions, random levels were obtained. All blood samples were processed and assayed within 1h of blood sample collection. Drug assays were performed using liquid chromatography-mass spectrometry via a previously published method [1]. All results are interpreted and reported by an infectious disease trained pharmacist within 24h of blood sample collection.

b. Results of therapeutic drug monitoring

On May 17th, 2024 (POD 85), upon the second recurrence of *Pseudomonas aeruginosa* (PA2091) bacteremia despite ongoing piperacillin-tazobactam administration, clinical decision was made to discontinue piperacillin-tazobactam and switch to cefepime 2g q8hrly for 2 weeks. On May 23th, 2024, in response to another breakthrough *P. aeruginosa* (PA2096) bacteremia, intravenous fosfomycin 8g q8hrly was added for its anti-biofilm activity [2-4]. TDM was also applied during the course of her subsequent treatment to optimize the pharmacokinetic and pharmacodynamic parameters. With standard doses of IV cefepime administered as a prolonged infusion over 4h, cefepime trough level of 11.7mg/L was deemed inadequate to eradicate bacteria with MIC of 8 mg/L as 40 mg/L (at 5X MIC) was the desired target.

Both cefepime 2g q8hrly and fosfomycin 8g q8hrly could not be administered through the outpatient antibiotic therapy (OPAT) service. As such, once infection was deemed under control and patient was fit for discharged, she was transitioned to high-dose IV piperacillin-tazobactam 4.5g q4hly monotherapy to facilitate drug administration through the OPAT as continuous infusion. However, both free piperacillin and tazobactam serum levels (at 60 – 63 mg/L and 13.1 to 13.6 mg/L, respectively) were deemed adequate, as they exceed 1) piperacillin concentration of 40mg/L or 5 times the piperacillin MIC value of 8mg/L reported for her bacterial isolate, and 2) tazobactam of 4mg/L. As the patient was keen for home, she was transitioned to IV piperacillin-tazobactam 21.6g/day (ran as a continuous outpatient antibiotic pump (of approximately 240ml) with supplemental doses of 4.5g self-administered once daily as short infusion. However, the antibiotic pump had large amount (approximately 40ml) of residual volume at the end of 24h, suggesting inadequate piperacillin-tazobactam delivery. This leads to inadequate piperacillin-tazobactam exposures (Table S1). Not surprisingly, a third recurrence of *P. aeruginosa* (PA 2111) bacteremia developed on high dose piperacillin-tazobactam on June 18, 2024, and this was picked up on surveillance blood cultures. Clinically she was asymptomatic with no localizing signs.

Subsequently, her outpatient antibiotic pump was switched to Easypump® elastomeric pump and the piperacillin-tazobactam dose was reduced to 18g/day for the antibiotic pump. This resolved the issue with residual volumes. In addition to Easypump® elastomeric pump administration, she continued to receive supplemental doses of piperacillin-tazobactam at 4.5g BD as short infusions to ensure adequate piperacillin-tazobactam exposure. However, she still could not attain adequate piperacillin-tazobactam exposure. Further dose increase was difficult due to logistical issues. We

were unable to secure pumps that could accommodate larger doses of piperacillin-tazobactam and the patient was reluctant to administer additional short infusions of piperacillin-tazobactam or to connect two OPAT pumps simultaneously.

Due to inability to attain adequate piperacillin-tazobactam exposure, oral levofloxacin 750mg daily was added to high dose piperacillin-tazobactam for additive interaction as guided by synergy E-test results. (See section 2 Synergy E-test below.) With the use of combination antibiotics, the MIC for both piperacillin and levofloxacin were reduced to 4mg/L (from 8 mg/L) and 2mg/L (from 4 mg/L), respectively. Hence, this reduced the therapeutic target she was required to achieve - free piperacillin target was reduced to 20mg/L while free tazobactam target remained at 4mg/L. Serial drug levels for piperacillin-tazobactam and levofloxacin are detailed in Table S1. With the reduction in piperacillin target, her piperacillin levels were deemed adequate but tazobactam levels remained subtherapeutic.

Table S1. Results of therapeutic drug monitoring

Sampling Date	Antibiotic Regimen	Free Drug Levels in Serum	Remarks
5 Jun 2024	IV Piperacillin-Tazobactam 4.5 g Q4H (each dose infused over 4 h) (i.e. continuous infusion)	Piperacillin: 60-63 mg/L (steady state random level)<br>Tazobactam 13.1-13.6 mg/L (steady state random level)	Target piperacillin level: 40 mg/L<br>Target tazobactam level: 4 mg/L
18 Jun 2024	IV Piperacillin-Tazobactam 21.6 g/day via OPAT pump + 4.5 g Q24H as bolus dose	Piperacillin 13.4 mg/L (steady state random level)<br>Tazobactam: 3.7 mg/L (steady state random level)	Significant residual volume in OPAT pump at the end of 24 h, indicating incomplete antibiotic administration and subtherapeutic levels
25 Jun 2024	IV Piperacillin-Tazobactam 21.6 g/day via OPAT pump + 4.5 g Q24H as bolus dose<br><br>With PO Levofloxacin 750 mg Q24H	Piperacillin: 14 mg/L (steady state random level)<br>Tazobactam: 3.4 mg/L (steady state random level)<br>Levofloxacin: 7.5 mg/L (peak level)	Target piperacillin level: 20 mg/L<br>Target tazobactam level: 4 mg/L<br>Target levofloxacin trough level: 2 mg/L<br>Levofloxacin was added based on synergy E-test results.<br>Unable to take levofloxacin trough levels in outpatient setting.
8 Jul 2024	IV Piperacillin-Tazobactam 18 g/day via Easypump® + 4.5 g Q12H as bolus dose<br><br>With PO Levofloxacin 750 mg Q24H	Piperacillin: 18 mg/L (steady state random level)<br>Tazobactam: 3.8 mg/L (steady state random level)<br>Levofloxacin: 3.1 mg/L (21h after dose)	Target piperacillin level: 20 mg/L<br>Target tazobactam level: 4 mg/L<br>Target levofloxacin trough level: 2 mg/L<br>Residual volume in OPAT pump resolved with the change in OPAT pump and dose;<br>Supplemental doses were continued to compensate for lower dose contained in OPAT pump<br>Unable to sample levofloxacin trough levels in outpatient setting
22 Jul 2024		Piperacillin: 28 mg/L (steady state random level)<br>Tazobactam: 5.7 mg/L (steady state random level)<br>Levofloxacin: 3.2 mg/L (18 h after dose)	
26 Aug 2024	IV Piperacillin-Tazobactam 18 g/day via Easypump® + 4.5 g Q12H as bolus dose<br><br>With PO Levofloxacin 500 mg Q24H	Piperacillin: 20 mg/L (steady state random level)<br>Tazobactam: 4.8 mg/L (steady state random level)<br>Levofloxacin: 1.8 mg/L (7h after dose)	Levofloxacin dose was reduced in view of complaints with nausea, but this reduced exposure to levofloxacin significantly.
25 Sep 2024	IV Piperacillin-Tazobactam 4.5 g Q4H (each dose infused over 1h)<br><br>With PO Levofloxacin 750 mg Q24H	Piperacillin: 84 mg/L (peak), 18mg/L (trough)<br>Tazobactam: 15 mg/L (peak), 3.7 mg/L (trough)<br>Levofloxacin: 2.3 mg/L (trough)	Regimen and TDM results during phage therapy

2. Synergy E-test.

a. Methods of synergy E-test

The patient's *P. aeruginosa* inoculum equal to 0.5 McFarland turbidity standard was prepared and 10 µl of the suspension was inoculated onto Mueller-Hinton agar plates. E test strips for piperacillin-tazobactam (with concentrations ranging from 0.016/4 to 256/4 mg/L) and levofloxacin (with concentrations ranging from 0.002 to 32 mg/L) were placed individually on each plate to determine their individual MICs. For synergy testing, piperacillin-tazobactam and levofloxacin E-test strips were placed perpendicularly on Mueller-Hinton agar plates. All plates were incubated at 35°C for 24 h under aerobic conditions. The MICs were determined from the point of intersection between the inhibition zone and the E-test strip. Fractional inhibitory concentration index (FICI) was calculated using a published formula to determine if synergism or additive activity was present [5].

b. Results of synergy E-test

The combination of piperacillin-tazobactam combination was found to be additive with FICI of 1. There were two-folds reductions in MICs observed for both piperacillin-tazobactam and levofloxacin. Piperacillin-tazobactam MIC decreased from 8/4 mg/L to 4/4 mg/L while levofloxacin MIC decreased from 4 mg/L to 2 mg/L.

3. Susceptibility profile of the patient's clinical *P. aeruginosa* isolates

a. Methods

Venous blood was inoculated into aerobic and anaerobic BD BACTEC™ bottles and transported to the microbiological laboratory. Blood cultures were processed employing the BD BACTEC™ FX system, where CO_2 produced reacts with a dye in the sensor, modulating the amount of light that is absorbed by a fluorescent material in the sensor. The light emitting diodes activates this fluorescent material and the level of fluorescence is measured by a photodetector. Analysis of the rate and amount of CO_2 increase enables the BD BACTEC™ FX machine to determine if the vial is positive. Positive bottles were gram stained and subsequently sub-cultured onto sheep blood agar (BD), MacConkey (Thermofisher Scientific), Chocolate agar (BD) plates. Mueller Hinton agar (BD) was used for disk diffusion testing and read after incubation at 35 ℃ for 16-18 hrs, as per CLSI M100 (34th edition) recommendations. Strains were identified by colony morphology, and results of the MALDI-TOF MS Microflex© (Bruker Daltonics Inc., Bremen, Germany).

b. Interpretative categories and disc diffusion zone diameters of four individual *P. aeruginosa* isolates based on Clinical Laboratory Standards Institute (CLIS) methods

Table S2. Interpretative categories and zone diameters of the *P. aeruginosa* isolates based on microbiology report using CLSI methods

Antibiotics	PA2081<br>20th Apr 2024	PA2091<br>17 May 2024	PA2096<br>23rd May 2024	PA2111<br>18th Jun 2024
Aztreonam (ATM)	S (27 mm)	S (25 mm)	S (27 mm)	S (23 mm)
Piperacillin/Tazobactam (PTZ)	S (28 mm)	S (24 mm)	S (28 mm)	S (23 mm)
Cefepime (CEF)	S (22 mm)	S (26 mm)	S (22 mm)	S (25 mm)
Ciprofloxacin (CIP)	I (20 mm)	S (30 mm)	I (23 mm)	S (34 mm)
Meropenem (MEM)	S (27 mm)	S (28 mm)	S (31 mm)	S (28 mm)

c. Minimum Inhibitory Concentrations (MICs) of four individual *P. aeruginosa* isolates and a four-bacteria mixture using microtiter MIC methods

Table S3. MIC of the four bacterial isolates based on laboratory report using microtiter MIC method*

Antibiotics	PA2081 20th Apr 2024	PA2091 17th May 2024	PA2096 23rd May 2024	PA2111 18th Jun 2024	4 bacteria mixture
Amikacin (AMK)	<=4	<=4	<=4	<=4	<=4
Aztreonam (ATM)	4	16	4	4	8
Ceftolozane/Tazobactam (C/T)	<=0.5/4	2/4	1/4	<=0.5/4	<=0.5/4
Colistin (CST)	1	0.5	1	1	1
Ceftazidime/Avibactam (CZA)	2/4	2/4	2/4	2/4	2/4
Doripenem (DOR)	<=0.5	<=0.5	<=0.5	<=0.5	<=0.5
Doxycycline (DOX)	>=32	16	>=32	8	>=32
Fosfomycin (FOF)	>=256	>=256	>=256	128	>=256
Imipenem (IPM)	<=0.5	<=0.5	<=0.5	<=0.5	<=0.5
Levofloxacin (LEV)	4	<=0.5	4	<=0.5	2
Meropenem (MEM)	<=0.5	<=0.5	<=0.5	<=0.5	<=0.5
Minocycline (MIN)	>=32	8	>=32	8	16
Polymyxin B (PMB)	1	1	1	1	1
Ampicillin/Sulbactam (SAM)	>=128/64	>=128/64	>=128/64	>=128/64	>=128/64
Tigecycline (TGC)	8	4	4	4	4
Temocilin (TMC)	>=16	>=16	>=16	>=16	>=16
Piperacillin/Tazobactam (PTZ)	8/4	64/4	8/4	32/4	16/4

Note: * The microtiter MIC method is described in the Method section of the main manuscript, and are expressed in mg/L.

4. Methods and results of whole genome analysis of the *P. aeruginosa* isolates

a. Methods

Total genomic DNA from *P. aeruginosa* isolates was extracted using the Qiagen DNeasy PowerSoil Pro Kit 47014 (Qiagen, Venlo, The Netherlands). For *P. aeruginosa* genome, sequencing libraries were prepared manually from 200 ng of purified DNA extracts using the Illumina DNA Prep (Illumina) in combination with the Illumina Nextera DNA CD Indexes (Illumina), as per the manufacturer’s instructions. Pooled libraries were sequenced using the MiniSeq platform with 2 × 150 bp chemistry as per the manufacturer's instructions (Illumina). Raw reads were trimmed, assembled, and annotated with Bacterial and Viral Bioinformatics Resource Center (BV-BRC, https://www.bv-brc.org/) tools with complementary analysis pipeline, including antimicrobial resistance and virulence factor genes identification with CARD and VFDB reference database. Multi-locus sequence typing was identified using PubMLST database (https://pubmlst.org/).

Defence systems in PA genomes were detected using PADLOC v2.0.0 with database version 2.0.0 [6] and DefenseFinder v.2.0.0 [7]. Only complete, previously characterized systems were reported, while Phage Defence Candidate (PDC) systems were excluded.

b. Results

All 4 isolates were classified as ST252 and harbored the same pattern of AMR genes, including the intrinsic β-lactamases $bla_{\text{OXA-50}}$ and $bla_{\text{PDC-3}}$; the multiple-drug resistance pump family Mex-Opr/Omp system, consisting of MexAB-OprM, MexCD-OprJ, MexEF-OprN, MexHI-OpmD, MexJK-OprM/OpmH, MexPQ-OpmE, MexVW-OprM, armA/B (also known as MexXY/AxyXY) with its regulator mexZ, and the TriABC-OpmH system. Isolates were also harbouring the acquired AMR genes including aminoglycoside transferase (*aph(3')-IIb*), chloramphenicol O-acetyltransferase (*catB7*), and fosfomycinase (*fosA*). Mutations or variants on specific genes to confer resistance were also found like the mutations in *gyrA* and *parC/E* can confer fluoroquinolone resistance, while modifications in *pmrA/B* altered cell wall charge, conferring polymyxin resistance.

Table S4. Results of whole genome analysis of the four *P. aeruginosa* isolates

**ID**	**PA2081**	**PA2091**	**PA2096**	**PA2111**
**Accession**	SRR32962967	SRR32962966	SRR32962965	SRR32962964
Genome size (bp)	7,130,020	7,130,041	7,061,677	7,129,787
GC (%)	65.6	65.6	65.7	65.6
CDS	7,043	7,051	6,919	7,059
ST	ST252			
AMR	*amrA, amrB, aph(3')-IIb, arnA, catB7, emrE, FosA, gyrA, mexA-S/V/W/Z, nalC/D, nfxB, opmD/E/H, oprD/J/M/N, oqxB, bla*$_{\text{OXA-50}}$*, parC/E, bla*$_{\text{PDC-3}}$*, phoP/Q, pmrA/B, triA/B/C*			
VF	*alg44/8, algA-L/P-R/U/W/X/Z, aprA, chpA-E, clpV1, dotU1, exoS/T/Y, exsA-E, fha1, fimT/U/V, fleN/Q/R/S, flgA-K/M,/flgN, flhA/B/F, fliA/E-R, fptA, fpvA, hcp1, hsiA1, hsiB1/vipA, hsiC1/vipB, hsiE1/F1/G1/H1/J1, icmF1/tssM1, lasA/B/I, lip1, mbtH-like, motA-D/Y, mucA-E/P, pchA-I/R, pcr1-4/D/G/H/R/V, phzA1* (×3), *phzB1* (×3), *phzC1/D1/E1/F1/G1/H/M/S, pilE-K/M-X/Y2, plcH, popB/D/N, ppkA, pppA, pscB-L/N, pscO-U, ptxR, pvcA-D, pvdA, pvdD* (×7), *pvdE-H, pvdI* (PA2081×5, PA2091×7,PA2096×7, PA2111×5,) *pvdJ* (×3), *pvdL-Q/S, rhlA-C/I, tagF/pppB, tagQ-T, toxA, tse1-3, vgrG1a/b, waaA/C/F/G/P, xcpA/pilD, xcpP-Z.*			
Prophages	No Virulence Factors<br>No AMR genes			

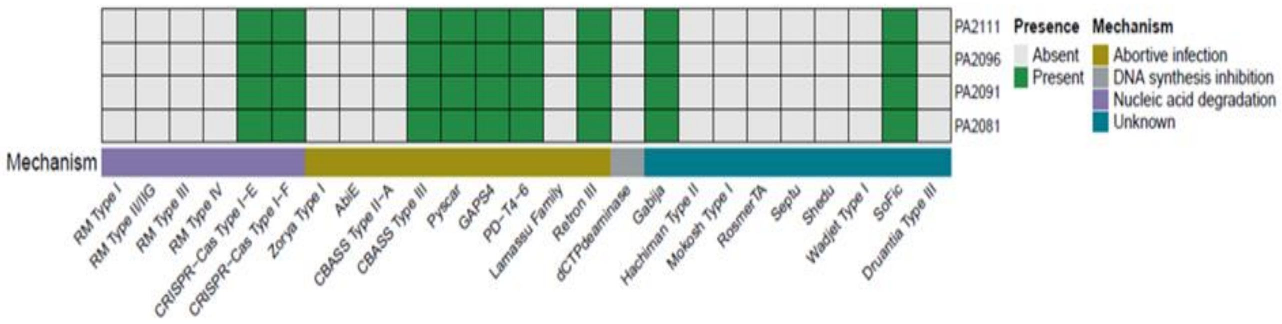

Figure S1. **Presence/absence matrix of defence systems identified in genomes clinical PA isolates from this study**. The list of most common 25 defence mechanisms was based on previous reports of distribution of defence system in clinical and environmental PA isolates [8, 9].

5. Phage genomic profiles

Table S5: Summary of genomic information of the three therapeutic phages used in this case

**Phage ID**	**PW21**	**KSY1a**	**P0413**
**Accession**	SRR32963843	SRR32963841	SRR32963842
**Length**	67,991	282,489	66,492
**GC Content**	55.4	36.9	55.7
**Order**	Caudovirales	Caudovirales	Caudovirales
**Genus**	*Myoviridae*	*Phikzvirus*	*Myoviridae*
**Species**	*Pbunavirus*	*Phikzvirus*	*Pbunavirus*
**Lifestyle**	virulent	virulent	virulent
**AMR Genes**	none	none	none
**Virulence Factors**	none	none	none

Table S6: Phage genomic passport of PW21

Assembly Information	
**# of Total Trimmed Reads**	8,815,884
**# of Mapped Read**	8,796,930
**% of Reads Mapped**	99.44%
**# of Variants with >1% Abundance**	3
**# Insertion Variants >50 bp**	0
**# of Contigs in Final Assembly**	13
**Linear of Circular Assembly?**	Linear
**Mean Coverage**	
**Genomic Information**	
**Genome Length**	67,991 bp
**GC Content**	55.35%
**Open Reading Frames**	105
**Hypothetical Proteins**	63
**Proteins with Functional Assignment**	42
**tRNA**	0
**Genus Prediction**	Pbunavirus
**Genomic Analysis**	
**# Antibiotic Resistance CDS**	0
**# Bacterial Virulence CDS**	0
**Closest Sequenced Relative**	PP716131
**# Attachment Sites**	0
**# of Integrases**	0
**Phage lifestyle Prediction**	Virulent
**% Reads Mapped to Host**	0%

Table S7: Phage genomic passport of KSY1a

**Assembly Information**	
**# of Total Trimmed Reads**	8,671,248
**# of Mapped Read**	8,643,226
**% of Reads Mapped**	99.71%
**# of Variants with >1% Abundance**	0
**# Insertion Variants >50 bp**	0
**# of Contigs in Final Assembly**	1
**Linear of Circular Assembly?**	Circular
**Mean Coverage**	2082.0x
**Genomic Information**	
**Genome Length**	282,489 bp
**GC Content**	37%
**Open Reading Frames**	362
**Hypothetical Proteins**	274
**Proteins with Functional Assignment**	88
**tRNA**	6
**Genus Prediction**	*Phikzvirus*
**Genomic Analysis**	
**# Antibiotic Resistance CDS**	0
**# Bacterial Virulence CDS**	0
**Closest Sequenced Relative**	NC_042060
**# Attachment Sites**	0
**# of Integrases**	0
**Phage lifestyle Prediction**	Virulent
**% Reads Mapped to Host**	0%

Table S8: Phage genomic passport of P0413

Assembly Information	
**# of Total Trimmed Reads**	8,838,856
**# of Mapped Read**	8,770,436
**% of Reads Mapped**	99.89%
**# of Variants with >1% Abundance**	1
**# Insertion Variants >50 bp**	0
**# of Contigs in Final Assembly**	1
**Linear of Circular Assembly?**	Linear
**Mean Coverage**	9256.3x
**Genomic Information**	
**Genome Length**	66,492 bp
**GC Content**	55.7%
**Open Reading Frames**	93
**Hypothetical Proteins**	56
**Proteins with Functional Assignment**	37
**tRNA**	0
**Genus Prediction**	Pbunavirus
**Genomic Analysis**	
**# Antibiotic Resistance CDS**	0
**# Bacterial Virulence CDS**	0
**Closest Sequenced Relative**	MT491206
**# Attachment Sites**	0
**# of Integrases**	0
**Phage lifestyle Prediction**	Virulent
**% Reads Mapped to Host**	6%

6. Transmission electron microscopy (TEM) images of 8 candidate therapeutic phages

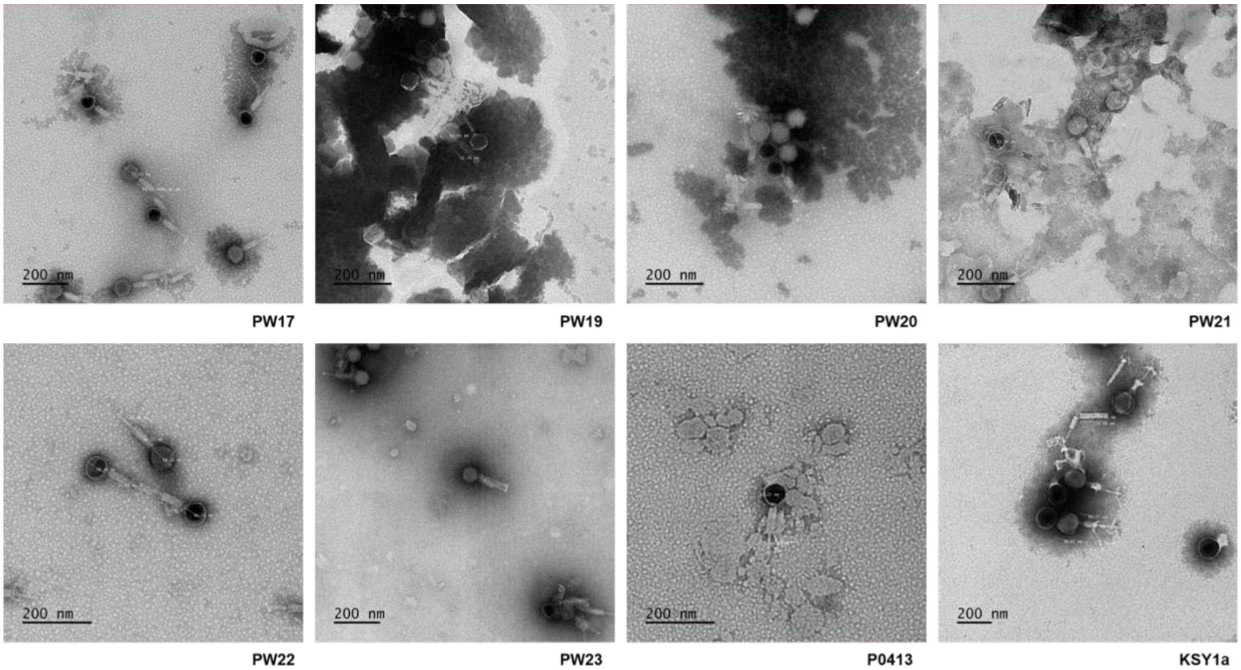

Figure S2. Transmission electron microscopy (TEM) images of bacteriophages PW21, KSY1a, and P0413, negatively stained with phosphotungstic acid. Scale bar: 200 nm. The TEM micrographs revealed head-tail structures. The phage capsid diameters are: KSY1a - 127 nm, P0413 - 66 nm, PW17 - 64 nm, PW19 - 66 nm, PW20 - 71 nm, PW21 - 67 nm, PW23 - 69 nm, and PW22 - 66 nm.

Methods:

Drops of phage suspension were placed on formvar/carbon supported copper grids (Electron Microscopy sciences, USA). Negative staining was performed with 2% phosphotungstic acid for 2 min. Grids were examined with a transmission electron microscope (JEOL 1400, Japan) and the images were acquired with a digital camera (Gatan, Ametek, USA).

7. Identification of suitable therapeutic phages

a. Phage susceptibilities via spot test

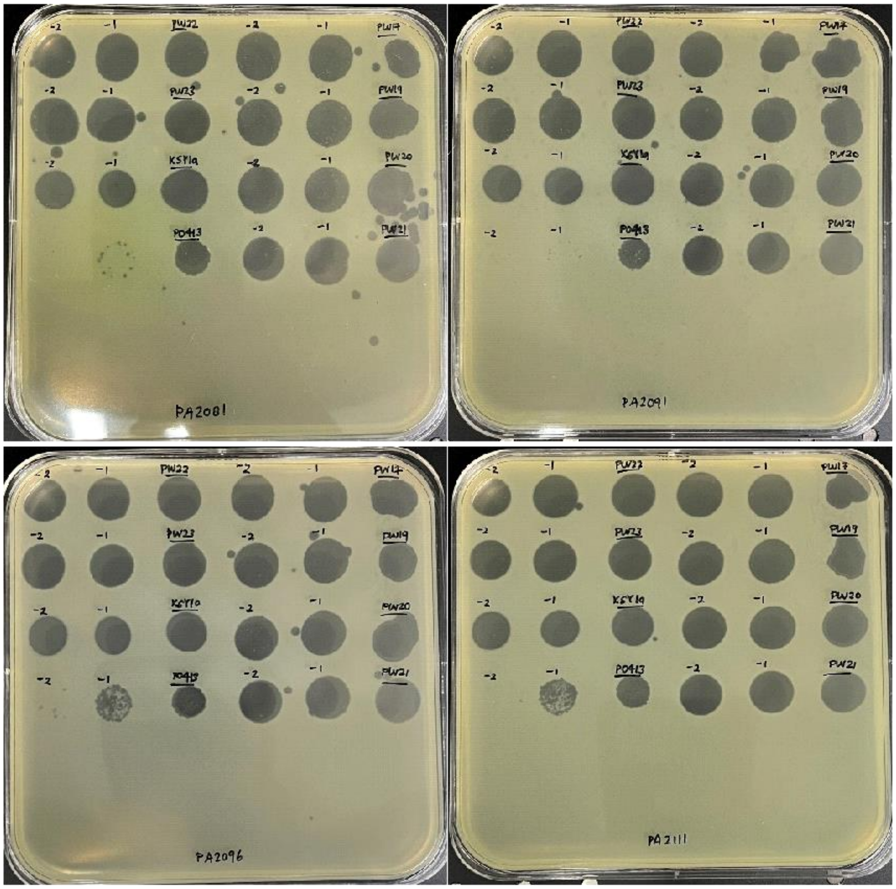

Figure S3. Patient bacteria isolates PA2081, PA2091, PA2096 and PA2111 susceptibility to serial dilutions of tested bacteriophages PW17, PW19, PW20, PW21, PW22, PW23, KSY1a and P0413. Eight distinct phages showed infectivity against the 4 *P. aeruginosa* isolates.

b. Antibiotics Micro-titre MICs assay when combined with phages

Table S9. Bacteria phage-antibiotic susceptibility profiles. Phage treatments that render at least 2-fold reduction in antibiotic MIC are labelled in green.

Antibiotics	4 bacteria mixture	+ PW20 PW21 KSY1a	+ PW21 PW23 KSY1a	+ PW21 KSY1a P0413	+ PW23 KSY1a P0413
Amikacin (AMK)	<=4	<=4	<=4	<=4	<=4
Aztreonam (ATM)	8	<=0.5	<=0.5	<=0.5	<=0.5
Ceftolozane /Tazobactam (C/T)	<=0.5/4	<=0.5/4	<=0.5/4	<=0.5/4	<=0.5/4
Colistin (CST)	1	<=0.25	<=0.25	<=0.25	<=0.25
Ceftazidime /Avibactam (CZA)	2/4	<=0.5/4	<=0.5/4	<=0.5/4	<=0.5/4
Doripenem (DOR)	<=0.5	<=0.5	<=0.5	<=0.5	<=0.5
Doxycycline (DOX)	>=32	<1	<1	<1	<1
Fosfomycin (FOF)	>=256	<16	<16	<16	<16
Imipenem (IPM)	<=0.5	<=0.5	<=0.5	<=0.5	<=0.5
Levofloxacin (LVX)	2	<=0.5	<=0.5	<=0.5	<=0.5
Meropenem (MEM)	<=0.5	<=0.5	<=0.5	<=0.5	<=0.5
Minocycline (MIN)	16	<=1	<=1	<=1	<=1
Polymyxin B (PMB)	1	<=0.25	<=0.25	<=0.25	<=0.25
Ampicillin/Sulbactam (SAM)	>=128/64	<=4/2	<=4/2	<=4/2	<=4/2
Tigecycline (TGC)	4	<=0.25	<=0.25	<=0.25	<=0.25
Temocilin (TMC)	>=16	<=0.5	<=0.5	<=0.5	<=0.5
Piperacillin/Tazobactam (TZP)	16/4	<4/4	<4/4	<4/4	<4/4

c. Kill kinetic studies of phages against patient clinical isolates

i). Single phage kill-kinetic against single bacteria isolate

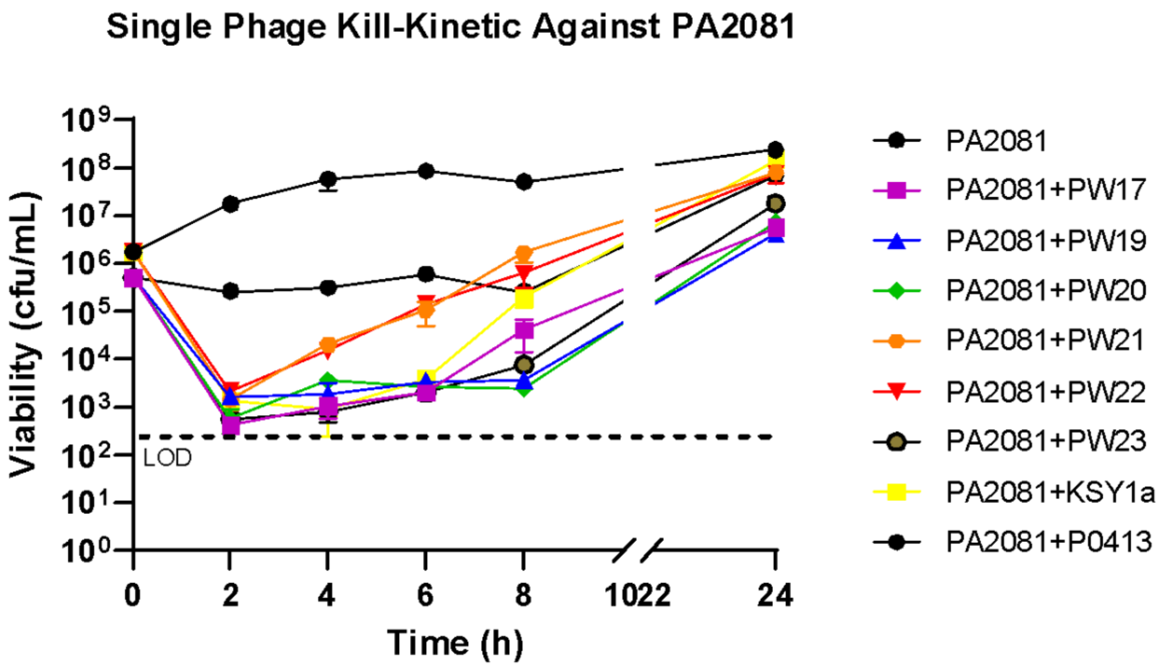

Figure S4: Time-kill kinetics of *P. aeruginosa* isolate PA2081 treated with eight bacteriophages candidates. The initial inoculum and final bacterial cell counts are indicated. LOD refers to limit of detection, which is 5 × 10^2.

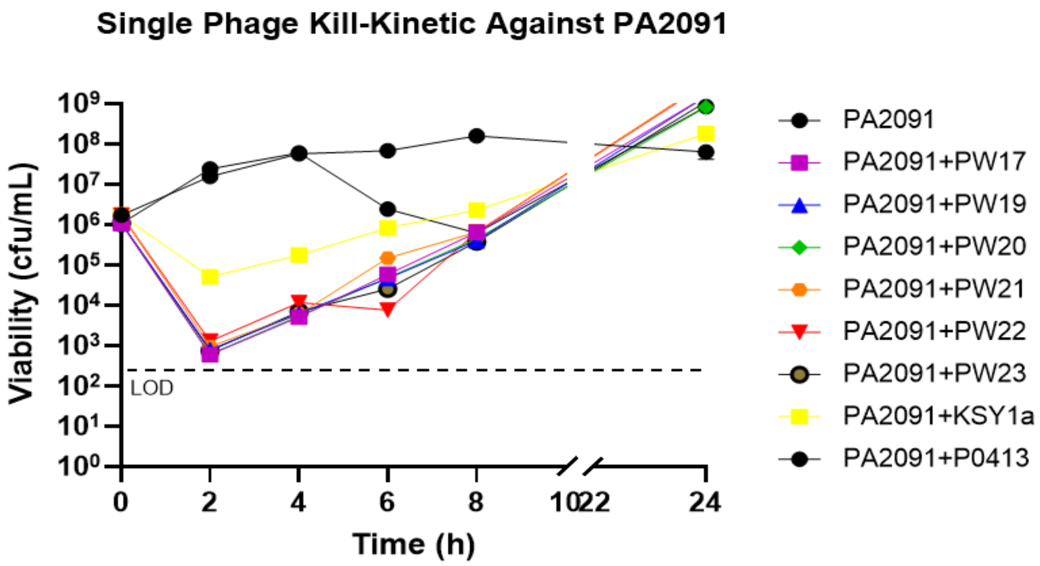

Figure S5: Time-kill kinetics of *P. aeruginosa* isolate PA2091 treated with eight bacteriophages candidates. The initial inoculum and final bacterial cell counts are indicated. LOD refers to limit of detection, which is 5 × 10^2.

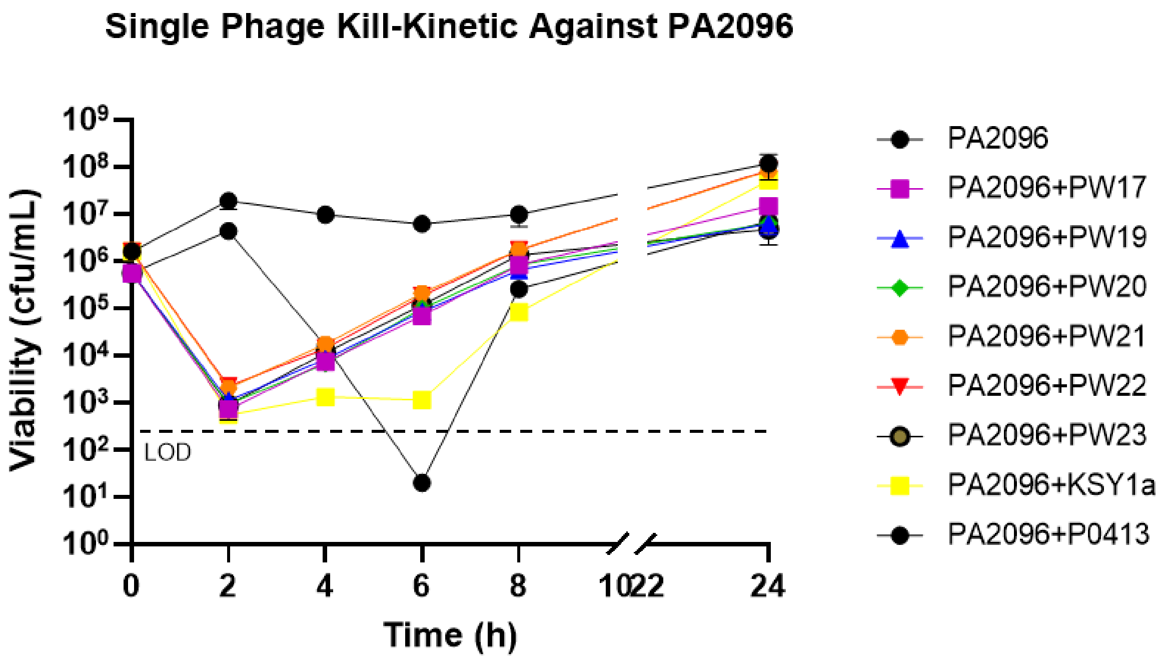

Figure S6: Time-kill kinetics of *P. aeruginosa* isolate PA2096 treated with eight bacteriophages candidates. The initial inoculum and final bacterial cell counts are indicated. LOD refers to limit of detection, which is 5×10^2.

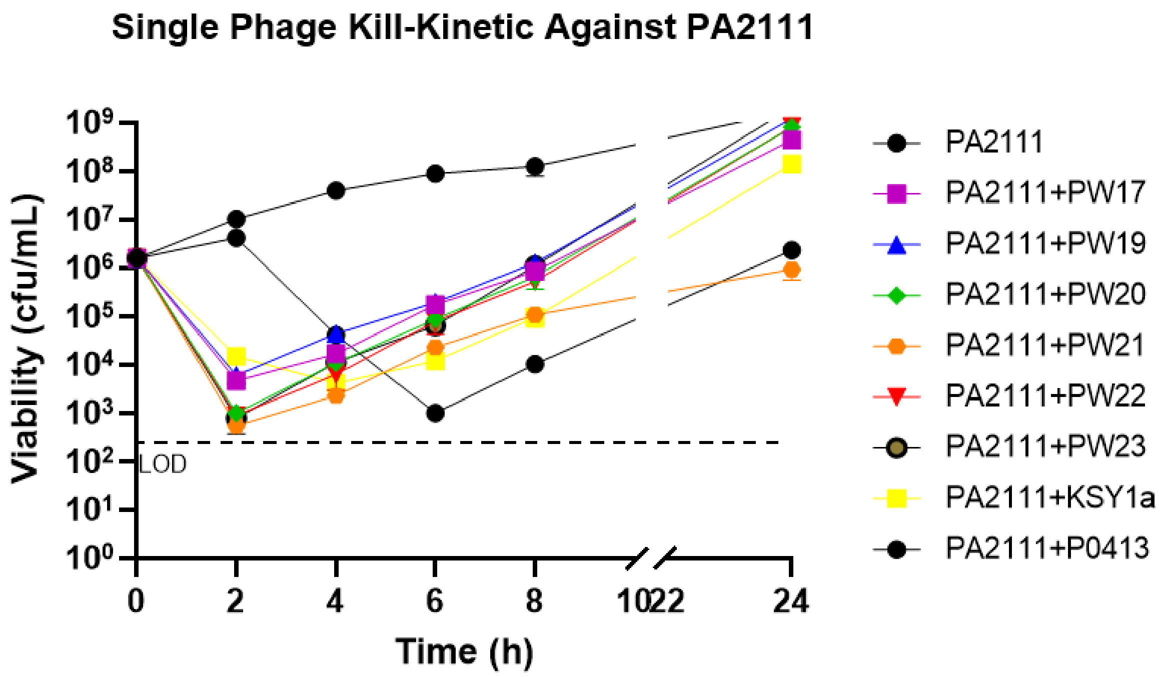

Figure S7: Time-kill kinetics of *P. aeruginosa* isolate PA2111 treated with the three individual phages. The initial inoculum and final bacterial cell counts are indicated. LOD refers to limit of detection, which is 5×10^2.

Bacteriophages PW20, PW21, PW23, KSY1a and P0413 are recommended for the following reasons:

PW20 and PW23 showed lower bacteria cell concentrations than other phages, consistently for PA2081, PA2091 and PA2096.

PW21 showed significantly lower bacteria cell concentrations for PA2111, the latest bacteria strain retrieved from patient blood culture.

KSY1a and P0413 showed very different killing patterns from the other phages for all bacterial isolates. KSY1a showed growth rebound later at 6h and 4h time point as compared to other phages for PA2096 and PA2111 respectively. P0413 always show initial bacteria growth before killing, especially significantly so for PA2096 and PA2111.

ii). <u>2-Phage cocktails kill-kinetic against 4-patient isolates combined.</u>

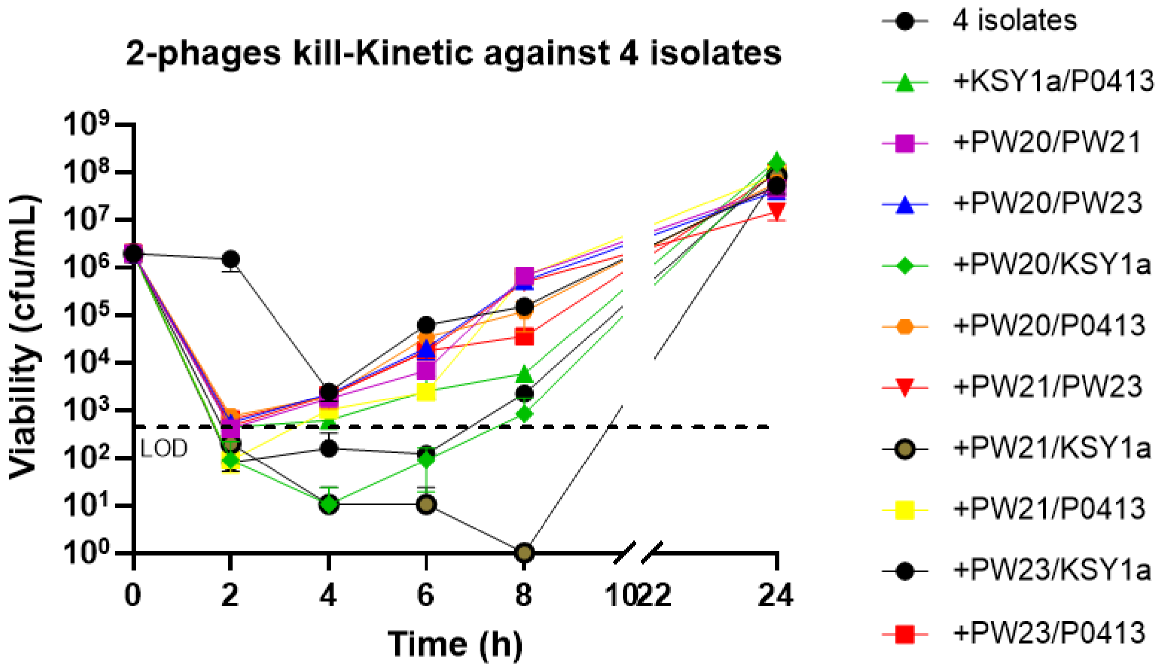

Figure S8: Time-kill kinetics (2-phages). 4-bacteria mixture of *P. aeruginosa* isolates PA2081, PA2091, PA2096, and PA2111 in the presence or absence (Control) of the two-phages cocktails (combinations of PW20, PW21, PW23, KSY1a and P0413). The initial inoculum and final bacterial cell counts are indicated. LOD refers to limit of detection, which is 5×10^2. The sharp initial drop in bacterial density observed in the control was likely attributable to sub-optimal growth conditions for this set of experiments.

iii). 3-Phage cocktails kill-kinetic against 4-patient isolates combined

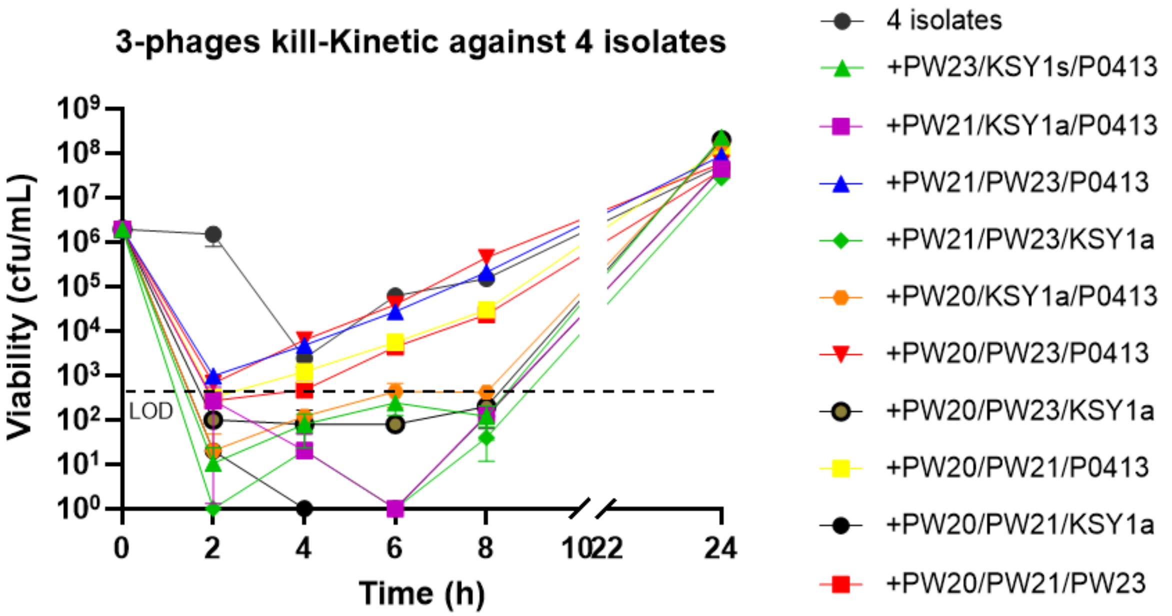

Figure S9: Time-kill kinetics (3-phages). 4-bacteria mixture of *P. aeruginosa* isolates PA2081, PA2091, PA2096, and PA2111 in the presence or absence (Control) of the three-phages cocktails (combinations of PW20, PW21, PW23, KSY1a and P0413). The initial inoculum and final bacterial cell counts are indicated. LOD refers to limit of detection, which is 5×10^2. The sharp initial drop in bacterial density observed in the control was likely attributable to sub-optimal growth conditions for this set of experiments.

The following 4 phage cocktails are recommended.

a. PW20 PW21 KSY1a
b. PW21 PW23 KSY1a
c. PW21 KSY1a P0413
d. PW23 KSY1a P0413

The rationale for recommending the above named cocktails is as follow:
PW20 PW21 KSY1a and PW21 KSY1a P0413 maintained bacterial killing for the longest time.
PW21 PW23 KSY1a had greatest initial bacterial killing.
All 4 cocktails showed slower bacterial regrowth.

d. Antibiotics kill kinetics against 4 patient clinical isolates combined

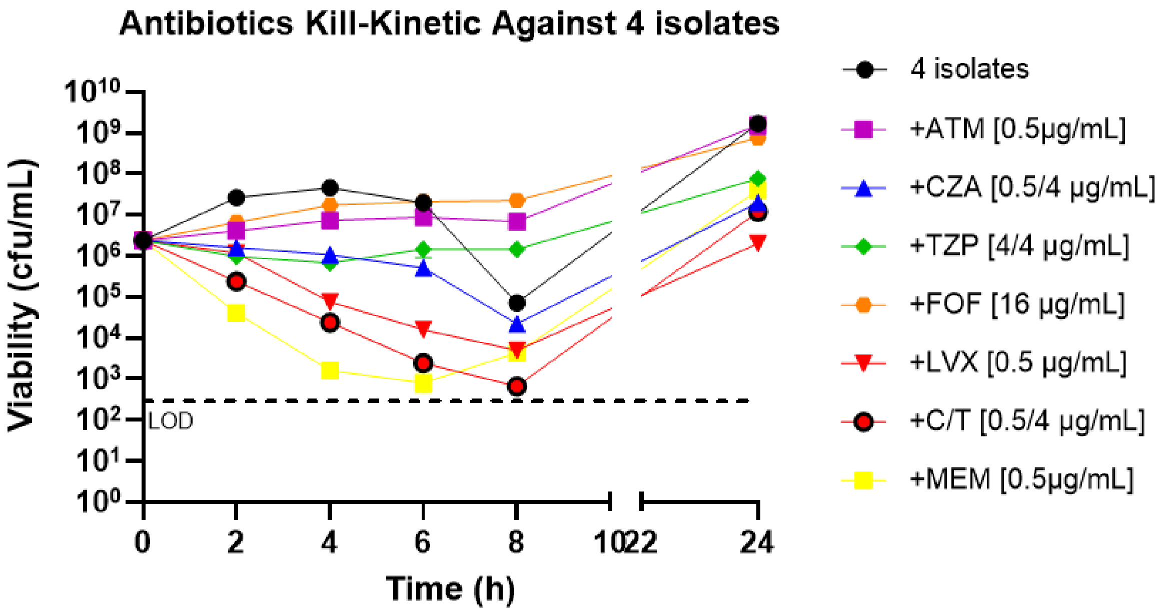

Figure S10: Time-kill kinetics (antibiotics). of a 4-bacteria mixture of *P. aeruginosa* isolates PA2081, PA2091, PA2096, and PA2111 in the presence or absence (Control) of selected antibiotics. The initial inoculum and final bacterial cell counts are indicated. LOD refers to limit of detection, which is 5×10^2. The sharp initial drop in bacterial density observed in the control was likely attributable to sub-optimal growth conditions for this set of experiments.

e. 3-Phage cocktails + antibiotics kill-kinetic against 4 patient isolates combined

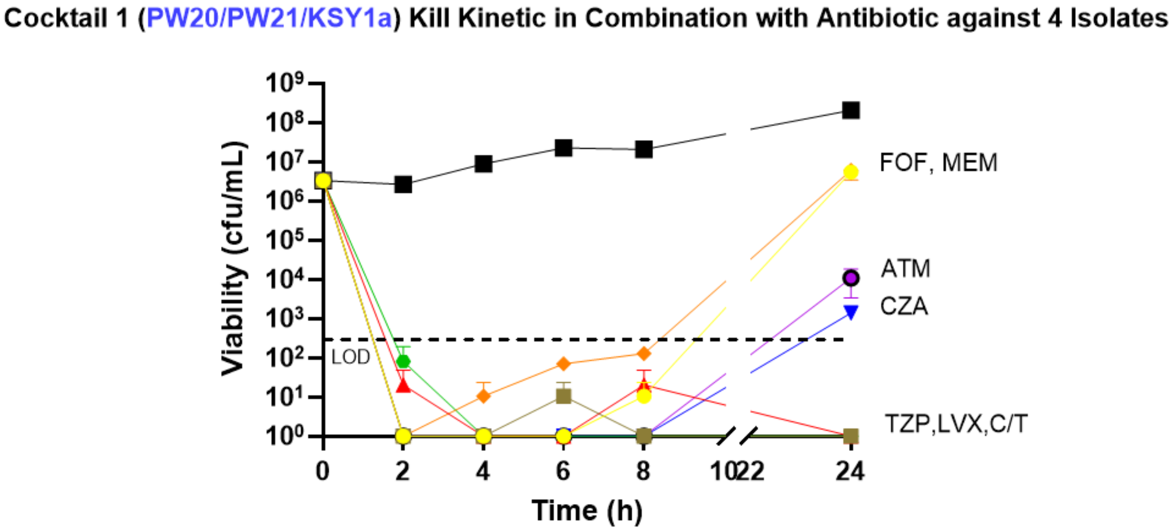

Figure S11: Time-kill kinetics (cocktail 1). 4-bacteria mixture of *P. aeruginosa* isolates PA2081, PA2091, PA2096, and PA2111 in the presence or absence (Control) of PW20, PW21 and KSY1a phage cocktail and selected antibiotics. The initial inoculum and final bacterial cell counts are indicated. LOD refers to limit of detection, which is 5×10^2.

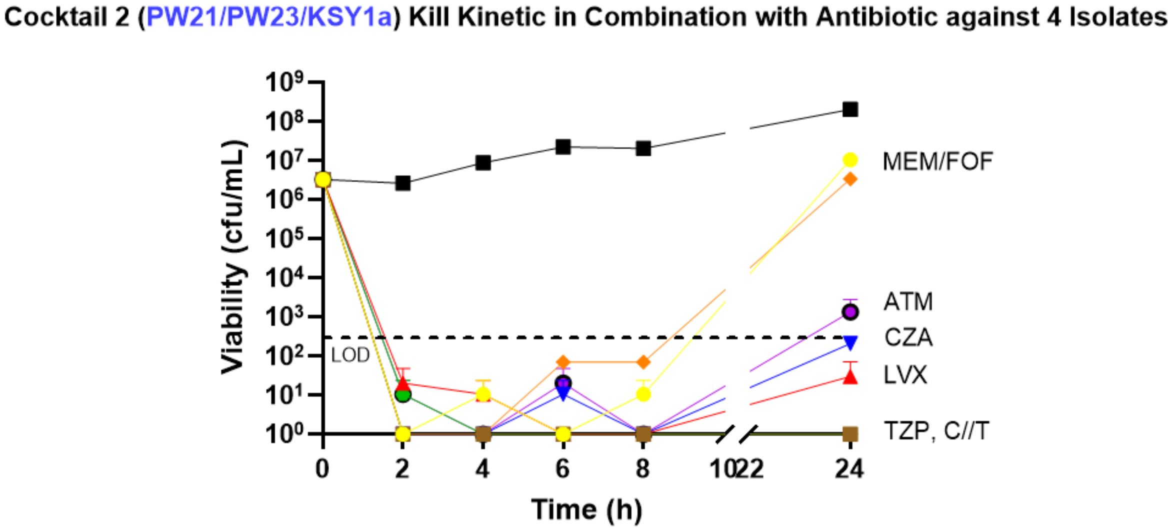

Figure S12: Time-kill kinetics (cocktail 2). 4-bacteria mixture of *P. aeruginosa* isolates PA2081, PA2091, PA2096, and PA2111 in the presence or absence (Control) of PW21, PW23 and KSY1a phage cocktail and selected antibiotics. The initial inoculum and final bacterial cell counts are indicated. LOD refers to limit of detection, which is 5×10^2.

Cocktail 3 (PW21/KSY1a/P0413) Kill Kinetic in Combination with Antibiotic against 4 Isolates

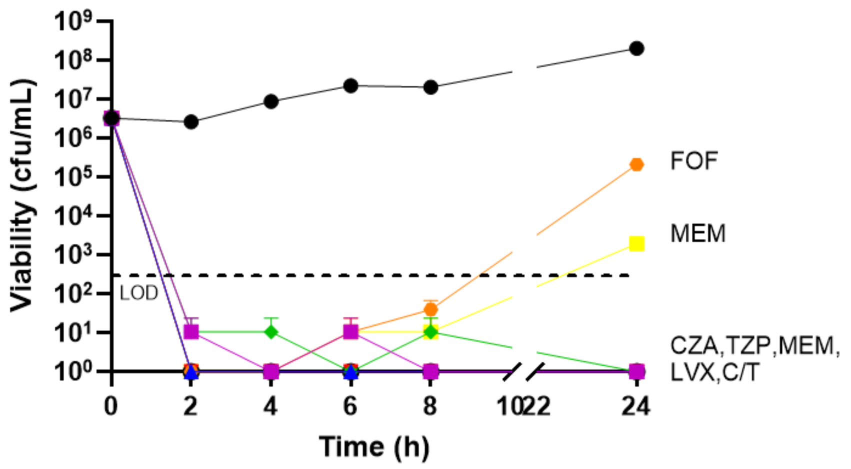

Figure S13: Time-kill kinetics (cocktail 3). 4-bacteria mixture of *P. aeruginosa* isolates PA2081, PA2091, PA2096, and PA2111 in the presence or absence (Control) of PW21, KSY1a and P0413 phage cocktail and selected antibiotics. The initial inoculum and final bacterial cell counts are indicated. LOD refers to limit of detection, which is 5×10^2.

Cocktail 4 (PW23/KSY1a/P0413) Kill Kinetic in Combination with Antibiotic against 4 Isolates

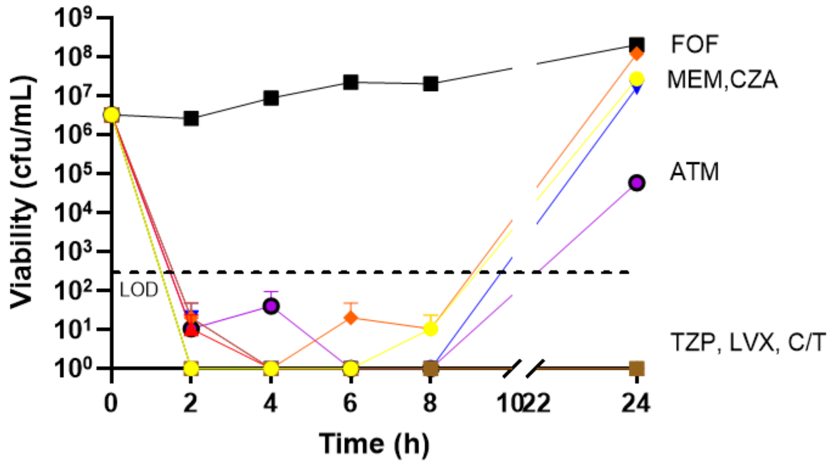

Figure S14: Time-kill kinetics (cocktail 4). 4-bacteria mixture of *P. aeruginosa* isolates PA2081, PA2091, PA2096, and PA2111 in the presence or absence (Control) of PW23, KSY1a and P0413 phage cocktail and selected antibiotics. The initial inoculum and final bacterial cell counts are indicated. LOD refers to limit of detection, which is 5×10^2.

Synergistic phage-Abx interactions consistently observed for phage cocktails with piperacillin/tazobactam, levofloxacin and ceftolozane/tazobactam.

PW21 KSY1a P0413 cocktail is recommended as it works well with most selected antibiotics.

f. **Single-phage and cocktail kill-kinetic at low MOI 1 against 4 patient clinical isolates combined.**

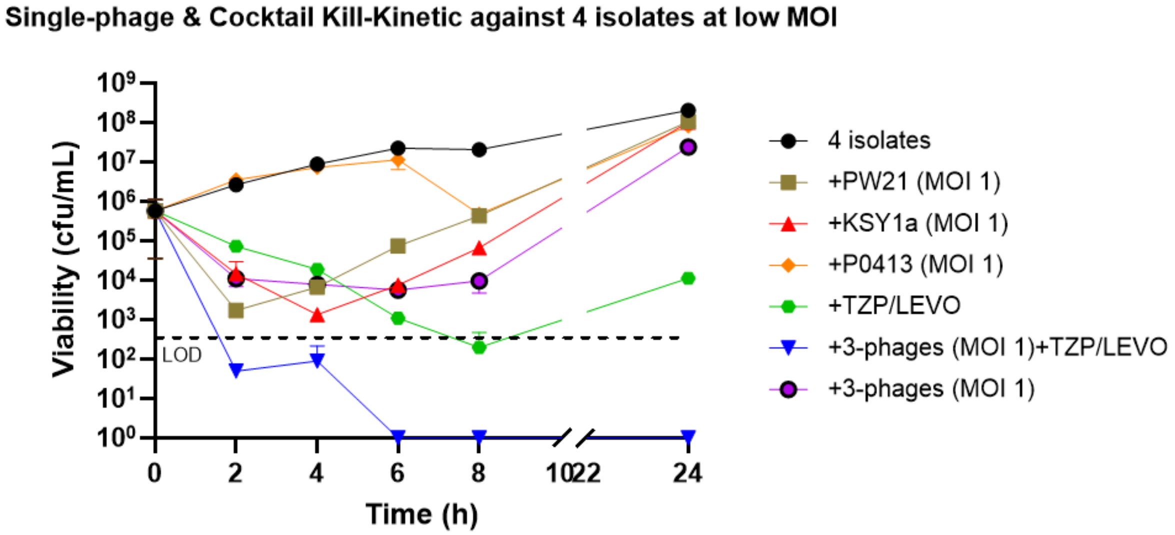

Figure S15: Time-kill kinetics at low MOI. 4-bacteria mixture of *P. aeruginosa* isolates PA2081, PA2091, PA2096, and PA2111 in the presence (phages at MOI 1 and/ or antibiotics) or absence (Control) of phages and antibiotics. The initial inoculum and final bacterial cell counts are indicated. LOD refers to the limit of detection, which is 5×10^2. The killing kinetics of bacteria by phages with higher MOI 100 and lower MOI 1 are similar.

8. Anti-Biofilm Activity

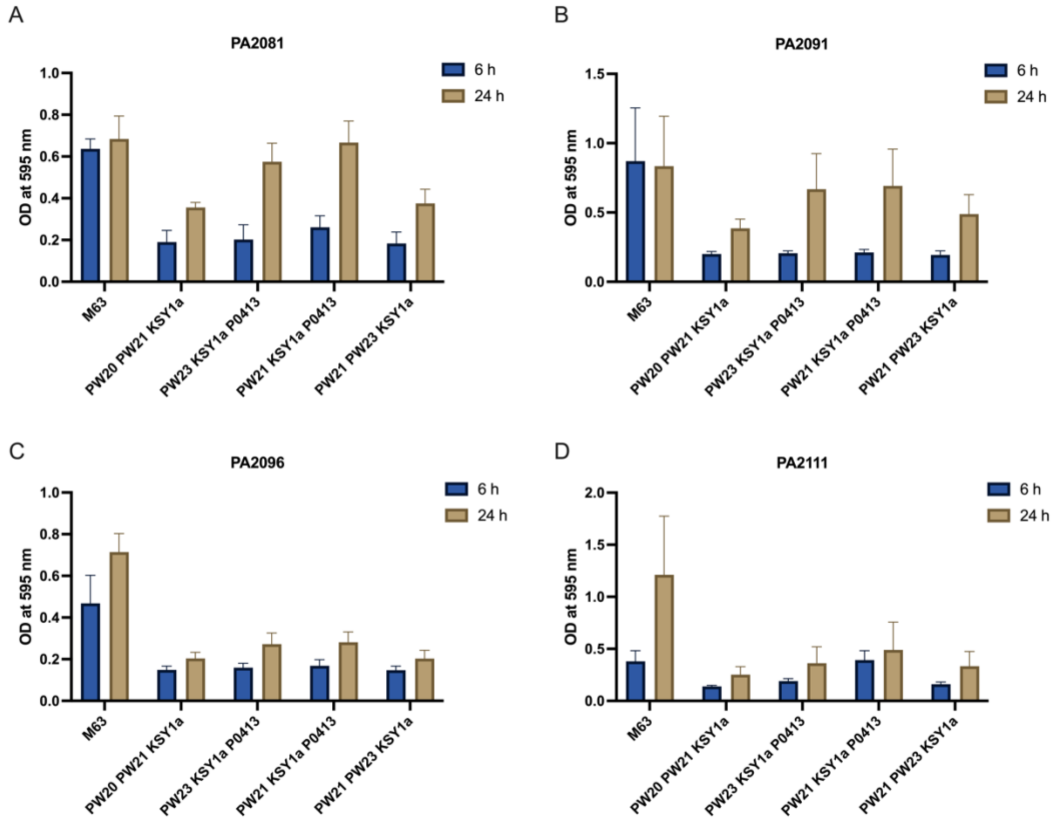

Figure S16: Biofilm dispersion assay with phage cocktails treatment on 6 h and 24 h biofilm formed from bacterial isolate PA2081 (A), PA2091 (B), PA2096 (C), and PA2111 (D) individually, M63 (with bacteria mixture) serves as control. M63 is a growth media commonly used for bacterial culture. Data are presented as mean values +/- SD (n=10 replicate wells).

We tested the ability of our phage cocktails to disperse PA2111 mature biofilms *in vitro*. Four different 3-phages cocktails were used to treat 6 h and 24 h-old biofilms followed by either crystal violet (CV) staining as a proxy for biofilm biomass, and colony forming units (CFU) enumeration as a measure of bacteria viability. All four cocktails showed propensity to disperse both young (6 h) and old (24 h) biofilms, albeit with different efficiency. Notably, all cocktails were able to reduce 24 h-biofilm biomass by up to 3 times. The reduction in biofilm-biomass correlated with a decrease in bacteria viability as illustrated by the reduction in CFU recovered from phage-treated biofilm (see Figure 4 in main article).

9. Role of the MexAB-OprM efflux pump in phage susceptibility

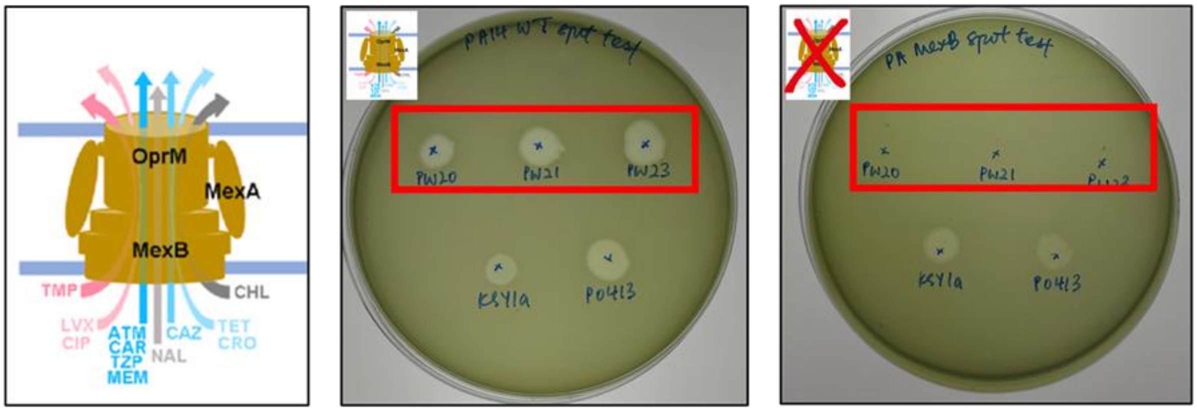

Figure S17. Role of the MexAB-OprM efflux pump in phage susceptibility. (Left) Schematic of the MexAB-OprM efflux pump in *Pseudomonas aeruginosa*, showing antibiotic substrates [10]. (Centre) Spot test on wild-type *P. aeruginosa* showing susceptibility to phages PW20, PW21, PW23, KSY1a and P0413 (red box). (Right) Spot test on a *mexB* mutant showing loss of susceptibility to PW20, PW21 and PW23 (red box), indicating that MexAB-OprM is required for infection by these phages. The *mexB* mutant has previously been reported by us [11] and is deficient in MexAB-OprM complex. We showed that the loss of MexAB-OprM resulted in a phenotypic trade-off, i.e. an acquired resistance to phage infection but an exacerbated sensitivity to antibiotics.

10. Single-phage escape bacteria mutant cross-resistance profiles

Table S10. Cross resistance profile of patients isolates after single-phage exposure.

	Phages				
Clinical Isolate Escape Mutants	PW20	PW21	PW23	KSY1a	P0413
PA2081 resistant to PW20	Resistant	Resistant	Resistant	Susceptible	Resistant
PA2081 resistant to PW21	Resistant	Resistant	Resistant	Susceptible	Resistant
PA2081 resistant to PW23	Resistant	Resistant	Resistant	Susceptible*	Susceptible*
PA2081 resistant to KSY1a	Susceptible	Susceptible	Susceptible	Resistant	Susceptible
PA2081 resistant to P0413	Susceptible*	Susceptible*	Susceptible*	Susceptible*	Resistant
PA2091 resistant to PW20	Resistant	Resistant	Resistant	Susceptible	Resistant
PA2091 resistant to PW21	Resistant	Resistant	Resistant	Susceptible	Resistant
PA2091 resistant to PW23	Resistant	Resistant	Resistant	Susceptible	Resistant
PA2091 resistant to KSY1a	Susceptible	Susceptible	Susceptible	Resistant	Susceptible
PA2091resistant to P0413	Resistant	Resistant	Resistant	Susceptible	Resistant
PA2096 resistant to PW20	Resistant	Resistant	Resistant	Susceptible	Resistant
PA2096 resistant to PW21	Resistant	Resistant	Resistant	Susceptible	Resistant
PA2096 resistant to PW23)	Resistant	Resistant	Resistant	Susceptible	Resistant
PA2096 resistant to KSY1a	Susceptible	Susceptible	Susceptible	Resistant	Susceptible
PA2096 resistant to P0413	Resistant	Resistant	Resistant	Susceptible	Resistant
PA2111 resistant to PW20	Resistant	Resistant	Resistant	Susceptible	Resistant
PA2111 resistant to PW21	Resistant	Resistant	Resistant	Susceptible	Resistant
PA2111 resistant to PW23	Resistant	Resistant	Resistant	Susceptible	Resistant
PA2111 resistant to KSY1a	Susceptible	Susceptible	Susceptible	Resistant	Susceptible
PA2111 resistant to P0413	Susceptible	Susceptible	Susceptible	Susceptible	Resistant

At the exposure of each bacterial isolate to a single phage in a kill-kinetic study, the phage escape bacteria mutant at 24-hr was subjected to spot tests by the 4 other phage candidates. * Indicate a reduction in lytic plaque size compared to wild-type bacteria. PW20, PW21 and PW23 infect the bacteria via the same mechanism of entry, when compared to KSY1a and P0413. Between KSY1a and P0413, both phage candidates infected the bacteria differently. Hence, the therapeutic phage cocktail (PW21+KSY1a+P0413) is likely to infect all the possible single-phage escape bacteria mutants, and the single phage constituent has a different cross-resistance profile.

11. Detection of Therapeutic Phages in Patient Blood

Method for PCR amplification of Phage DNA from blood samples:

PCR amplification was performed directly on phage lysates or patient's serum without prior DNA extraction. Detection sensitivity was assessed using ten-fold serial dilutions of phage lysates (10^7–10^1 PFU/mL) as templates. Primer specificity was evaluated using lysates of non-target phages at 10^6 PFU/mL. Mixed-template reactions with all 3 phages in 1:1:1 ratio were included to assess whether the assay could reliably detect the target phage in the presence of other phages, to mimic complex biological samples such as patient serum. Finally, patient serum samples collected on days 0, 8, and 15 were tested using the optimised PCR protocol. Each reaction contained 12.5 µL of Q5 High Fidelity 2x Master Mix (New England Biolabs), 0.5 µM of forward and reverse primers (table below), 2.5 µL of phage lysate as template and nuclease-free water to a final volume of 25 µL. PCR cycling was with a prolonged initial denaturation step at 98 °C for 3 min to lyse viral capsids and release viral DNA for amplification, followed by 35-40 cycles of denaturation at 98 °C for 10 s, annealing at 68°C for 30 s, and extension at 72 °C for 30 s, with a final extension at 72 °C for 2 min. Amplicons were separated by electrophoresis on a 1.5% agarose gel stained with Sybr Safe (Life Technologies) at 90 V for 70 min. A 100bp DNA Ladder (New England Biolabs) was used as a reference and visualised using an iBright 1500 gel imager (Invitrogen).

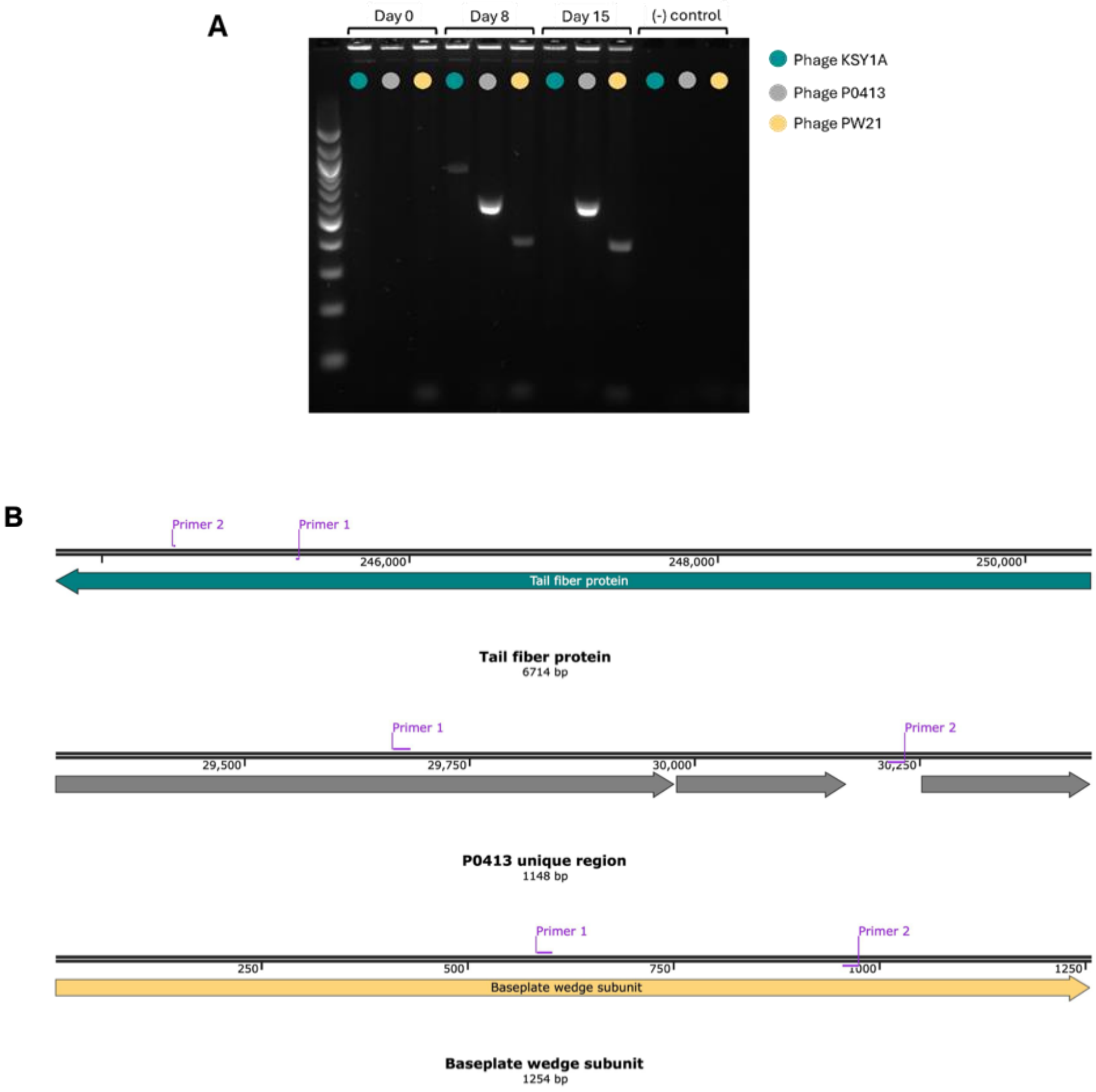

Figure S18: Detection of Therapeutic Phages in Patient Blood. A) PCR detection of target phages in patient serum samples. Lane M: 100bp DNA Ladder (New England Biolabs). Lanes 2–4: day 0 serum tested with primers specific for phages KSY1A (teal), P0413 (grey), and PW21 (yellow), respectively. Lanes 5–7: day 8 serum tested with t same primer sets. Lanes 8–10: day 15 serum tested same three primer Lanes 11–13: negative controls for each primer set. **B)** Selected regions for PCR targets are highlighted in teal, grey and yellow. Primers are indicated in purple. Forward (F) and reverse (R) primers used for PCR detection are indicated by arrows at their respective genomic positions. Expected size of amplicon is indicated in the primers table (Table S11) below.

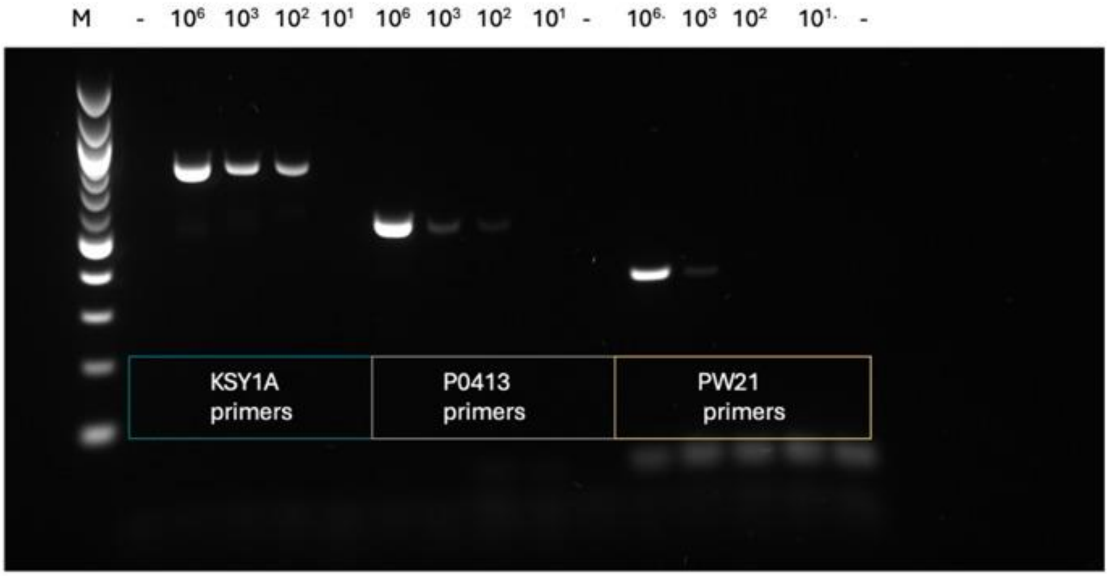

Figure S19. PCR optimization for detection of phages in blood samples. A) PCR amplification with primers specific for phage KSY1A. B) PCR amplification with primers specific for phage P0413. C) PCR amplification with primers specific for phage PW21. Lane M: DNA ladder. Lanes 1-2: PCR products from non-target phages (10^6 PFU/mL) to verify primer specificity. Lanes 3–10: KS1a serial dilutions from 10^7 to 10^1 PFU/mL. Lane N: negative control (no template) showing no amplification.

Table S11: Target gene or genomic region, expected PCR product length, and sequences of forward and reverse primers. Details are shown for the three phages used in this case.

Phage	Target gene or region	PCR product length	Forward primer (5'->3')	Reverse primer (5'->3')
KSY1A	Tail fiber protein 250419:243706 bp	819 bp	GCGCGTCCAGATTGC TAATG	TCAGTGGGTGCCCTA CCTAA
P0413	Unique region 29240:30352 bp	569bp	GGTGCGCGAAAATA CTCGTC	CGCTTCGGGATTCGA TGAGA
PW21	Baseplate wedge unit 28215:29468 bp	391bp	TCAATCGTCTGGCGA TCCAA	GAGGAAGAGCCCTGC TGAAC

Supplementary references

1. Goh, K.K.-K., et al., *Quantification of Fosfomycin in Combination with Nine Antibiotics in Human Plasma and Cation-Adjusted Mueller-Hinton II Broth via LCMS.* Antibiotics, 2022. **11**(1): p. 54.
2. Díez-Aguilar, M. and R. Cantón, *New microbiological aspects of fosfomycin.* Rev Esp Quimioter, 2019. **32 Suppl 1**(Suppl 1): p. 8-18.
3. Roussos, N., et al., *Clinical significance of the pharmacokinetic and pharmacodynamic characteristics of fosfomycin for the treatment of patients with systemic infections.* Int J Antimicrob Agents, 2009. **34**(6): p. 506-15.
4. Slade-Vitković, M., et al., *In Vitro Antibiofilm Activity of Fosfomycin Alone and in Combination with Other Antibiotics against Multidrug-Resistant and Extensively Drug-Resistant Pseudomonas aeruginosa.* Pharmaceuticals, 2024. **17**(6): p. 769.
5. Odds, F.C., *Synergy, antagonism, and what the chequerboard puts between them.* J Antimicrob Chemother, 2003. **52**(1): p. 1.
6. Payne L.J., et al., Identification and classification of antiviral defence systems in bacteria and archaea with PADLOC reveals new system types, *Nucleic Acids Research*, 8 Nov 2021. 49(19): 10868-78.
7. Tesson F. et al. Systematic and quantitative view of the antiviral arsenal of prokaryotes. Nat. Commun. 2022. 13: p. 2561
8. Burke, K.A.; Urick, C.D.; Mzhavia, N.; Nikolich, M.P.; Filippov, A.A. Correlation of *Pseudomonas aeruginosa* Phage Resistance with the Numbers and Types of Antiphage Systems. *Int. J. Mol. Sci.* **2024**, *25*, 1424.
9. Ana Rita Costa *et al.* Accumulation of defense systems in phage-resistant strains of *Pseudomonas aeruginosa.Sci. Adv.2024,* **10**,eadj0341.
10. Xu Z, Li M, Li Y, Cao H, Miao L, Xu Z, Higuchi Y, Yamasaki S, Nishino K, Woo PCY, Xiang H, Yan A. Native CRISPR-Cas-Mediated Genome Editing Enables Dissecting and Sensitizing Clinical Multidrug-Resistant P. aeruginosa. Cell Rep. 2019 Nov 5;29(6):1707-1717.e3.
11. Ho P, Dam LC, Koh WRR, Nai RS, Nah QH, Rajaie Fizla FBM, Chan CC, Aung TT, Goh SG, Fang Y, Lim Z, Koh MG, Demott M, Boucher YF, Malleret B, Gin KY, Dedon P, Moreira W. Screening of the PA14NR Transposon Mutant Library Identifies Genes Involved in Resistance to Bacteriophage Infection in *Pseudomomas aeruginosa*. Int J Mol Sci. 2024 Jun 26;25(13):7009.
